# The Effect of Non-Alcoholic Fatty Liver Disease on Weight Loss and Resolution of Obesity-Related Disorders After Bariatric Surgery

**DOI:** 10.1007/s00268-023-07153-8

**Published:** 2023-09-25

**Authors:** Thaer S. A. Abdalla, Anastasios D. Giannou, Ahmed S. A. Abdalla, Jakob R. Izbicki, Anna Dupreé, Oliver Mann, Stefan Wolter

**Affiliations:** Department of General, Visceral and Thoracic Surgery, At the University Hospital Hamburg-Eppendorf, Martinistrasse 52, 20251 Hamburg, Germany

## Abstract

**Background:**

Patients undergoing bariatric surgery have a high incidence of non-alcoholic fatty liver disease (NAFLD). However, the effect of NAFLD or non-alcoholic steatohepatitis (NASH) on the weight loss and resolution of obesity-related disorders is a matter of debate.

**Methods:**

In this study, we compare the long-term outcomes after bariatric with the presence of NAFLD in the liver biopsy at the time of surgery.

**Results:**

The follow-up was available for 226 out of 288 patients. The mean follow-up time was 24.9 (± 13.6) months. The baseline histology showed that 112 patients (38.9%) had no NASH, 70 (24.3%) were borderline, and 106 (36.8%) had NASH. At follow-up, the mean BMI dropped from (52 ± 10.2) to (36.6 ± 8) kg/m ^2^. Excess weight loss (EWL) was similar in all NAFLD groups. Type 2 diabetes mellitus dropped from 35.7 to 11.4%, hypertension from 65.6 to 36.7%, hyperlipidemia from 62.3 to 33%, and obstructive sleep apnea from 37.5 to 14.9%. Only hyperlipidemia was significantly associated with NASH compared to the groups with no NASH or borderline NASH (*p* value = 0.002 and *p* value = 0.04, respectively) during the first two years of follow-up.

**Conclusion:**

The beneficial effects of bariatric surgery are evident across all patients with NAFLD. Patients with NASH have comparable outcomes regarding weight loss and resolution of obesity-related comorbidities.

**Supplementary Information:**

The online version contains supplementary material available at 10.1007/s00268-023-07153-8.

## Introduction

Obesity is one of the greatest public health challenges of the twenty-first century [1]. In Germany, the prevalence of obesity, defined as a body mass index (BMI) ≥ 30kg/m^2^, is more than 22% [2].

On top of causing huge physical and psychological disability, obesity is the cause of many severe comorbidities like type II diabetes mellitus (T2DM), hypertension, obstructive sleep apnea syndrome (OSAS), and non-alcoholic fatty liver disease (NAFLD) [3, 4].

NAFLD is considered to be the hepatic manifestation of metabolic syndrome and is defined as the accumulation of fat in the hepatocytes without a history of alcohol consumption. It ranges from steatosis hepatis without inflammation to non-alcoholic steatosis hepatitis (NASH), which leads to fibrosis and eventually cirrhosis [5]. Since its first description in 1980, NAFLD has become the most common chronic liver disease worldwide [6]. Currently, it is estimated that the prevalence of NAFLD is as high as one billion [7]. Consequently, it is one of the most common causes of liver transplantation [8]. NASH has also been linked to the development of hepatocellular carcinoma (HCC) [9]. Therefore, early-stage NASH represents a group of patients that is most likely to benefit from preventive and therapeutic strategies aiming to limit disease progression.

Given the substantial morbidity associated with NASH, efforts were exerted to distinguish NASH from simple steatosis. For this purpose, several biomarkers, score systems, and imaging methods have been attempted. However, the current non-invasive techniques cannot identify NASH with complete certainty. Thus, liver biopsy is still considered the gold standard to diagnose NASH [10, 11].

Owing to the high incidence of NAFLD in obesity, which can be as high as 90% [12], many bariatric centers perform a routine liver biopsy during surgery to identify patients with asymptomatic advanced disease and offer early therapeutic interventions and appropriate disease surveillance [13]. Bariatric surgery has proven to be the most effective way to treat obesity and improve obesity-related comorbidities like T2DM, arterial hypertension, and OSAS. Despite the strong association between NAFLD and obesity, the role of bariatric surgery in NAFLD is still not clear. Much even less, is the effect of NAFLD on the success of bariatric surgery, providing long-term weight loss and resolution of comorbidities. The result of the recent studies has been inconclusive [14–16]. Our study aims to assess the significance of NAFLD on long-term weight loss, resolution of comorbidities, and late postoperative complications after bariatric surgery.

## Materials and methods

### Patient selection

Using a prospective database, we identified patients who had an intraoperative liver biopsy due to abnormal liver appearance during bariatric surgery. The indication for primary surgery was according to S3 German Guidelines [17]. Only patients undergoing SG or RYGB were included. All patients undergo preoperatively a rigorous psychiatric evaluation. In case of active substance abuse, such as alcohol or drug abuse, a bariatric operation will not be performed. Therefore, all patients included in this study were non-alcoholic.

Anthropometric measurements, presence, and resolution of obesity-related diseases, and laboratory studies were collected preoperatively and at follow-up. The ethics committee approved the clinical database. We obtained informed consent from all patients.

### Liver histology

A wedge-shaped resection of the left lobe of the liver was performed at the end of bariatric surgery in case of abnormal macroscopic appearance of the liver. All histologic specimens were reviewed by our pathologist. The liver biopsies were classified according to the NAS score. A NAS score over 5 is diagnostic for NASH [18]. According to the NAS Score, NAFLD can be divided into three groups: NAS <  (no NASH), NAS 3–4 (borderline NASH), and NAS ≥ 5 (NASH).

### Postoperative care and follow-up plan

In our center, follow-up is done 3, 6, 12, 18, and 24 months after surgery and then once yearly. During the follow-up, serial weight measurements and clinical examinations are done. Complete blood count, chemistry, kidney function test, liver function test, lipid profile, A1C, glucose, iron studies, vitamin B12, vitamin B1, vitamin D, calcitriol, PTH, and vitamin A are measured.

Postoperatively, patients should follow a customized nutritional plan. We recommend the daily intake of ursodeoxycholic acid 250 mg as prophylaxis for symptomatic gallstone disease for 6 months or till the end of the rapid weight loss period as well as the intake of a proton pump inhibitor e.g., pantoprazol 40mg for 6 months.

As part of daily nutrition, we recommend the intake of multivitamin preparations, 1g calcium, 60–90 g proteins. It is also recommended that premenopausal women avoid pregnancy in the first 2 years after the operation. Parenteral contraception methods are recommended. Osteodensitometry is recommended for all patients after 2 years [17].

### Statistical analysis

Statistical analysis was carried out using *IBM SPSS ver. 24 (Armonk, N.Y., USA)*. For categorical variables, Chi-square or Fisher exact tests were to show differences between groups. A *p value* ≤ of 0.05 was considered significant.

The analysis of the quantitative variables was performed using the Wilcoxon test for comparisons, before and after surgery. The Kruskal–Wallis test was used to compare the differences between these variables according to liver histology groups. Lastly, the late outcome of patients with NASH (NAS >  = 5) was assessed according to operation (SG vs. RYGB) using the Mann–Whitney test. For additional analyses, the applied statistical test is given in the corresponding part of the results section.

## Results

### Patient characteristics at baseline

Using our prospective database, we retrospectively identified 306 patients, in whom a liver biopsy was conducted at the time of surgery. Two hundred and eighty-eight subjects were included. Ten patients were excluded because they underwent other types of bariatric operations. Two patients were excluded due to other forms of liver disease (one patient with primary biliary cirrhosis, and one patient with hepatitis). Three patients refused enrollment and three patients were excluded due to inadequate liver biopsy. No patient had a history of alcohol abuse.

Follow-up was available for 226 out of 306 (73.8%) patients. The mean follow-up period was 24.9 months (± 13.6). One hundred and forty-nine patients (51.7%) underwent SG, while 139 (48.3%) patients underwent RYGB. During the follow-up, two patients died, one patient died of advanced osteosarcoma and the other patient died of myocardial infarction.

We observed a female predominance with 69.4%. The mean age was 43.4 years (± 11.96). The mean preoperative BMI was 52 (± 10.2) kg/m^2^. Patient demographics are shown in Table 1.

**Table 1:** Patient Demographics

	Preoperatively	Follow-up
Patients, *n*	288	236
Female sex, *n (%)*	200 (69.4%)	167 (72.9%)
Mean age, *y*	43	
Mean BMI, *kg/m*^*2*^	52 ± 10.2	36.6 ± 8
Mean weight, *kg*	151.5 ± 32	105.5 ± 24.4
EWL, *kg*		59.1 ± 25.9
Diabetes mellitus II, *n (%)*	103 (35.7%)	25 (11.4%)
Hyperlipidaemia, *n (%)*	179 (62.3%)	69 (33%)
Hypertension, *n (%)*	180 (65.6%)	79 (36.7%)
OSAS,* n (%)*	108 (37.5%)	32 (14.9%)
GERD,* n (%)*	45 (15.6%)	9 (4.2%)

*n*(%); number of subjects (Percentage), *BMI* Body mass index, *OSAS* obstructive sleep apnea, *GERD* gastroesophageal reflux disease, *y* years

The histology showed that 112 patients (38.9%) had no NASH, 70 (24.3%) were borderline, 106 (36.8%) had NASH and 11 (3.8%) patients had newly diagnosed asymptomatic liver cirrhosis. Histological parameters are shown in Table 2.

**Table 2:** Histological parameters at the time of surgery

Histological variables	*n* =	Percentage %
*NAFLD score*		
No NASH	112	38,9%
Borderline NASH	70	24,3%
NASH	106	36,8%
Total	288	100%
*Grade of fibrosis*		
0	171	59.4%
1	90	31.3%
2	11	3.8%
3	5	1.7%
4	11	3.8%
Total	288	100%
*Presence of cirrhosis*		
No Cirrhosis	277	96,2%
Cirrhosis	11	3,8%
Total	288	100%

*n* number of subjects, % Percentage, *NASH* non-alcoholic steatohepatitis

### Weight loss and resolution of comorbidities at follow-up concerning NAFLD

At the follow-up, the mean excess weight loss (%EWL) in our study group was 59.1% and the mean BMI was 36.2 (± 7) kg/m^2^. Patients with NASH tended to have a higher BMI, however without statistical significance between groups (*p* = *0.056).* Regarding postoperative weight, the NASH group weighed significantly more compared to the no NASH and borderline NASH groups, with a *p* value of 0.02 and 0.053, respectively (Table 3). 61.6% of the patients showed an excess weight loss above 50%. The complete remission of at least one comorbidity was found in 69% of the patients.

**Table 3:** Relation of NAFLD to anthropometric measurements, obesity-related comorbidities, and late postoperative complications at follow-up

	No NASH	BD-NASH	NASH	n	*P*	Pair-wise comparison
Weight preoperatively	147.3	146.8	159	288	0.012*	NASH versus No NASH (*P* = *0.008)* NASH versus BD NASH (*P* = *0.029)* No NASH versus BD NASH (*P* = *0.895)*
BMI preoperatively	50.7	50.4	53.4	288	0.066*	
Weight at follow-up	102.8	102.3	110.9	229	0.043*	NASH versus No NASH (*P* = *0.020)* NASH versus BD NASH (*P* = *0.053)* No NASH versus BD NASH (*P* = *0.980)*
BMI at follow-up	35.6	35	37.6	229	0.056*	
EWL	62.1	60.5	54.7	229	0.160*	
T2DM				218	0.669	NASH versus No NASH (*P* = *0.004)* NASH versus BD NASH (*P* = *0.020)* No NASH versus BD NASH (*P* = *0.872)*
*Present*	9	8	8			
*Not present*	80	46	67			
Hypertension				215	0.492	
*Present*	32	17	30			
*Not present*	57	37	42			
Hyperlipidemia				203	0.008	
Present	22	14	33			
*Not present*	62	37	35			
GERD				213	0.360	
*Present*	2	2	5			
*Not present*	84	52	68			
OSAS				214	0.398	
*Present*	12	6	14			
*Not present*	77	47	58			
Mobility				213	0.175	
*Improved*	57	43	60			
*Worse*	7	3	4			
*Unchanged*	22	8	9			
Arthrosis				211	0.100	
*Improved*	37	29	46			
*Worse*	5	1	2			
*Unchanged*	44	23	24			
Reflux				214	0.323	
*Improved*	17	11	13			
*Worse*	0	1	2			
*Unchanged*	70	42	58			

*p* value according to the χ2 test for categorical variables. *p value according to the Kruskal–Wallis Test. BD; Borderline

**Fig. 1:**
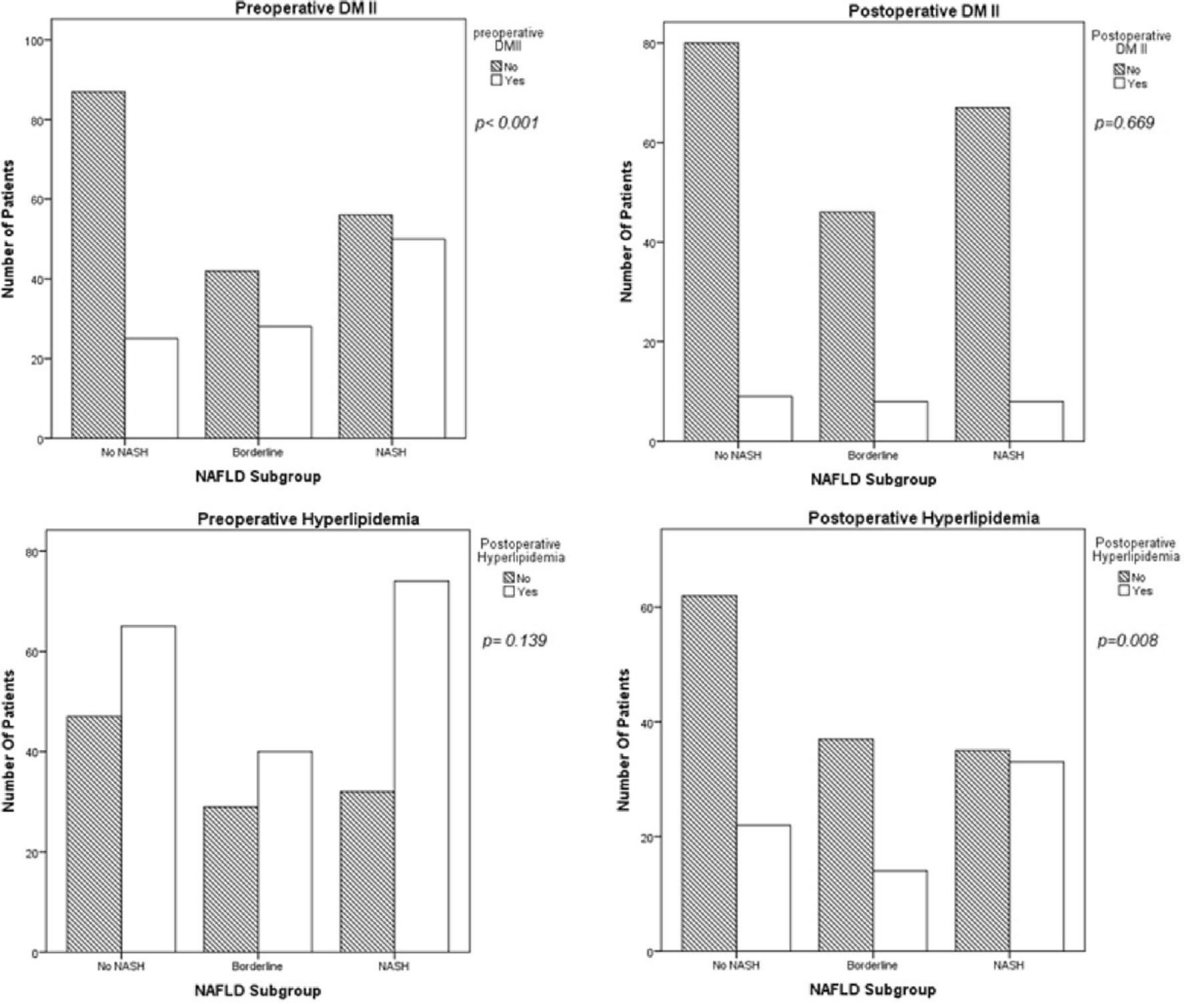
Prevalence of obesity-related comorbidities preoperatively and at follow-up. *P* value according to the *χ*2 test

The percentage of patients with preoperative T2DM was 35.7%, with hypertension 65.6%, with hyperlipidemia (LDL ≥ 100 in non-diabetics and ≥ 70 in diabetics) 62.3%, and with obstructive sleep apnea 37.5%. At follow-up, these percentages dropped to 11.4, 36.7, 33, and 14.9%, respectively. Only hyperlipidemia was associated with the NASH group compared to the no NASH and borderline NASH groups*,* with a *p* value *of* 0.002 and 0.04, respectively. Interestingly, this association was not present in patients (*n* = 120) with longer follow-up times > 24 months (median 36 ± 11 months) (NASH vs No NASH *p* = 0.259 and NASH vs. Borderline NASH, *p* = 0.169). All other obesity-related disorders were not relevant to NASH at follow-up (Table 3), (Fig. 1).

All biochemical parameters improved at follow-up in our cohort (Table 4). However, lower HDL, higher triglyceride, and cholesterol levels were associated with NASH at follow-up, with a p value of 0.03, 0.04, and 0.03*,*, respectively*.* Higher LDL levels tended to be higher in the NASH group, but levels of significance were not observed (*p value* = *0.057).* This effect was not present in patients (*n* = 120) after two years of follow-up (p value of 0.122, 0.254, 0.345, 0.98, respectively).

**Table 4:** Laboratory parameters at baseline and follow-up and their relation to NASH at follow-up

Laboratory Parameters	Baseline	Follow-up	Before/After p-value	Association to NAFLD at follow-up. *P*	Pair-wise comparison
A1C, *%*	6.4 ± 1.4	5.5 ± 1	< 0.001	0.41	
AST, *U/L*	25.9 ± 18	22.5 ± 24.3	< 0.001	0.270	
ALT, *U/L*	33.4 ± 20	25.8 ± 27.1	< 0.001	0.140	
GGT, *U/L*	68.8 ± 80	29.3 ± 30	< 0.001	0.890	
CRP, m*g/dl*	13.5 ± 11	7 ± 6	< 0.001	0.170	
LDL, *mg/dl*	105 ± 32	93.9 ± 33	< 0.001	0.057	
HDL, *mg/dl*	43.8 ± 12	59.3 ± 18	< 0.001	0.030	NASH versus No NASH (P = 0.001) NASH versus BD NASH (P = 0.017) No NASH versus BD NASH (P = 0.497)
Triglyceride, *mg/dl*	209 ± 108	149.8 ± 99	< 0.001	0.041	NASH versus No NASH (*P* = *0.035)* NASH versus BD NASH (*P* = *0.202)* No NASH versus BD NASH (*P* = *0.286)*
Cholesterol, *mg/dl*	189 ± 38	180.5 ± 36.2	0.006	0.039	NASH versus No NASH (*P* = *0.309)* NASH versus BD NASH (*P* = *0.011)* No NASH versus BD NASH (*P* = *0.098)*
Folic acid, *µg/l*	7.9 ± 5	10.4 ± 6.1	< 0.001	0.980	
Vitamin B12, *ng/l*	522.5 ± 234	564.5 ± 297	0.29	0.690	
Vitamin D, *µg/l*	12.2 ± 7	25.8 ± 11.6	< 0.001	0.013	NASH versus No NASH (*P* = *0.004)* NASH versus BD NASH (*P* = *0.086)* No NASH versus BD NASH (*P* = *0.849)*

*p* indicates significance according to the Wilcoxon Test, A1C (normal < 6.5%); ALT, alanine aminotransferase (normal 10–35 U/L); AST, aspartate aminotransferase (normal 10–35 U/L); CRP, C-reactive protein (normal < 5 mg/L); serum triglycerides (normal, 70–180 mg/dL); total cholesterol (normal 150–200 mg/dl); HDL, high density lipoprotein (normal 45–65 mg/dl); LDL, low density lipoprotein (normal < 150 mg/dl); folic acid (normal 4.6–18.7 µg/l); Vitamin B-12 (normal 270-730 ng/l); 1,25(OH)-D-Vitamin D (calcitriol) (normal > 30 pg/ml); BD, Borderline; Wilcoxon test compared numerical variables in the whole population before and after surgery. The Kruskal–Wallis Test compared the relation of NAFLD to the laboratory parameters at follow-up. *p* value ≤ 0.05 is considered significant

### Sleeve Gastrectomy (SG) versus Roux-Y Gastric Bypass (RYGB) in NASH patients

The NASH group was further analyzed according to the type of operation (SG vs. RYGB). There was no difference in BMI and the prevalence of T2DM, hypertension, and hyperlipidemia at follow-up.

Further analysis showed lower levels of ALT, AST, and GGT in the SG group compared to RYGB, with a *p* value of 0.029, 0.06, and 0.04, respectively, and lower levels of LDL and cholesterol in the RYGB (*p* value 0.002 and *p* value 0.01, respectively).

## Discussion

The results of this study show that patients with NASH have comparable outcomes regarding long-term weight loss compared to other NAFLD subgroups, Furthermore, bariatric surgery resulted in the resolution of obesity-related comorbidities like diabetes mellitus, hypertension, and OSAS after bariatric surgery.

With the increasing obesity pandemic worldwide, NAFLD has become the most common liver disease worldwide [7]. The acknowledged efficacy of bariatric surgery on obesity and its related comorbidities has caused more and more bariatric surgeries to be performed every year [19]. Not surprisingly, bariatric surgeons encounter frequently patients with NASH and advanced liver disease at the time of operation. Therefore, understanding the consequences of surgery in this group of patients is of utmost importance.

The incidence of NAFLD in bariatric patients is high. The incidence of NAFLD in our cohort was 74.6%. The histology showed a high percentage of NASH 35.9% and liver cirrhosis 3.8% in our patients, which is consistent with other published series [20]. Although patients with NASH are at increased risk of HCC [9], no case with HCC was detected in our cohort at follow-up.

The efficacy of bariatric surgery in our cohort is in line with the current literature. In our cohort, a mean excess weight loss (%EWL) of 59.1% was documented, a percentage that is comparable to other studies [21]. This positive effect was evident across all NAFLD groups.

As expected, NASH patients had significantly higher A1C preoperatively compared to the borderline NASH (*p* value 0.05*)* and no NASH (p value 0.001*)* groups. This finding is in line with the results from Cazzo et al. [22] who reported that T2DM was highly associated with NASH. However, at follow-up, there was no difference among the groups (*p* value 0.4) with a mean A1C of 5.5%. Likewise, there was a significant improvement of other obesity-related disorders like hypertension and OSAS, which was not related to the presence of NASH in the histology.

NAFLD is atherogenic and is characterized by hypertriglyceridemia, high LDL, and low HDL [23]. At baseline, hyperlipidemia was related equally to all NAFLD groups. However, at follow-up, hyperlipidemia was mostly associated with NASH compared to the no NASH (*p* value  = 0.002) and the borderline NASH (*p* value  = 0.04) groups. The persistence of high non-HDL-Cholesterol in NASH patients after bariatric surgery was also reported by Corey et al*.* [24]. This observation suggests that long-term pharmacological therapy on top of bariatric surgery may be needed in patients with biopsy-proven NASH.

The current NAFLD/NASH treatment includes pharmaceuticals and weight loss, mostly achieved with lifestyle modification. However, this has poor effects in patients with a body mass index > 35kg/m^2^ [25]. The role of bariatric surgery as a therapeutic strategy in the case of NASH is yet to be determined. A Cochrane meta-analysis could not show a specific impact of bariatric surgery on NASH due to insufficient studies [26]. The SPLENDOR study which included 1158 patients with histologically proven NASH, showed that patients undergoing bariatric surgery had, compared with nonsurgical management, a significantly lower risk of incident major adverse liver outcomes and major adverse cardiovascular events [27], In our cohort, biochemical analyses showed a general improvement in all NAFLD groups at follow-up. However, a second biopsy is needed to confirm these changes.

Previous studies have suggested that, NASH was associated with worse outcome after bariatric surgery. Hypothetically, the association of NASH with insulin resistance, can affect appetite regulation, energy expenditure and fat storage, making it harder to lose weight [28]. Moreover, the association of NASH with chronic inflammation can as well lead to impaired fat metabolism [29, 30] and reduce the ability to burn fat after surgery. Lastly, NASH has been associated with reduced metabolic rate [31], which in turn may lead to impaired weight loss postoperatively. Nevertheless, our data show that bariatric surgery can be performed in patients with NASH at comparable outcomes to other NAFLD groups. The presence of steatosis or fibrosis was associated with 50% excess weight loss at follow-up. Therefore, our results disagree with observations of other published series [14, 15], this highlights the independent impact of surgery and continuous diatery consultation on weight loss after bariatric surgery. Furthermore, we were not able to identify any preoperative parameter that was associated with adequate excess weight loss on follow-up (Supplementary Table 1). However, when taking only patients with NASH into consideration, our results showed lower levels of ALT, AST, and GGT at follow-up in the SG group compared to RYGB, with a *p value* of 0.029, 0.06, and 0.04, respectively, and lower levels of LDL and cholesterol in the RYGB (p value 0.002 and p value 0.01, respectively) compared to SG at follow-up. However, more data are needed regarding the type of surgery for NASH and liver fibrosis resolution.

Finally, several limitations must be addressed for the proper interpretation of our results. First, this is a retrospective study that was conducted using a prospective bariatric database at tertiary bariatric institution in Germany. In addition, a second liver biopsy at follow-up was only conducted by a small number of patients, therefore, the effect of bariatric surgery on liver histology could not be assessed in this study. Moreover, we used wedge liver resection as our biopsy method, therefore, the presence of broad band fibrosis in the specimen cannot be excluded. Another limitation is that liver biopsy was only conducted in patients with macroscopic abnormal liver appearance, so that prevalence of NAFLD in case of normal liver appearance could not be evaluated. Lastly, in our center preoperative imaging studies like ultrasound or elastography for the detection of NAFLD and liver fibrosis are not part of the routine work-up, therefore we limited the biopsy to patients with macroscopic pathological appearance during the study period. Nevertheless, to our knowledge this is one of the largest studies assessing the effect of histology proven NAFLD on weight loss after bariatric surgery.

## Conclusion

The beneficial effects of bariatric surgery are evident across all patients with NAFLD. The weight loss and metabolic benefits after bariatric surgery are independent of NASH.

### Supplementary Information

Below is the link to the electronic supplementary material.

Supplementary file1 (DOCX 13 kb)

## Data Availability

The datasets used during the current study are available from the corresponding author on reasonable request.
