# Supplementary material for: The Effect of Non-Alcoholic Fatty Liver Disease on Weight Loss and Resolution of Obesity-Related Disorders After Bariatric Surgery
Source: World J Surg. 2023 Sep 25;47(12):3281–8. doi: 10.1007/s00268-023-07153-8 (PMC10694115; doi:10.1007/s00268-023-07153-8)
Supplement: Supplementary file 1 — Supplementary file1 (DOCX 13 kb) [file 268_2023_7153_MOESM1_ESM.docx]

Supplementary Table 1: Association of different perioperative parameters on 50% excess weight loss after bariatric surgery.

| **Variables** | **Failure** | **Success** | **p-value** |
| --- | --- | --- | --- |
| Sex  female  male | 63  25 | 104  37 | 0.129 |
| T2DM  No  Yes | 54  34 | 97  44 | 0.248 |
| Hypertension  No  yes | 34  54 | 42  99 | 0.167 |
| Hip-Arthrosis  No  Yes | 81  7 | 135  6 | 0.239 |
| Type of Surgery  RYGB  Sleeve | 40  48 | 71  70 | 0.470 |
| NAFLD  No NASH  BD NASH  NASH | 33  22  33 | 59  35  47 | 0.769 |
| Liver Fibrosis  No  Yes | 48  40 | 85  56 | 0.392 |
| BMI  <50 *kg/m^2^*  ≥50 *kg/m^2^* | 40  48 | 67  74 | 0.761 |
| Triglyceride > 150 *mg/dl*  Yes  No | 23  63 | 49  90 | 0.184 |
| LDL > 100 *mg/dl*  No  Yes | 42  41 | 52  78 | 0.129 |
| HDL > 60 *mg/dl*  No  Yes | 77  9 | 124  15 | 0.939 |
| CRP > 5 *mg/dl*  No  Yes | 14  74 | 28  112 | 0.438 |

Legend: p-value according to the χ2 test
